# Effects of Polydopamine Microspheres Loaded with Silver Nanoparticles on *Lolium multiflorum*: Bigger Size, Less Toxic

**DOI:** 10.3390/toxics9070151

**Published:** 2021-06-29

**Authors:** Xinrui Wang, Hongyong Luo, Weihua Zheng, Xinling Wang, Haijun Xiao, Zhen Zheng

**Affiliations:** 1 School of Chemistry and Chemical Engineering, Shanghai Key Laboratory of Electrical Insulation and Thermal Ageing, Shanghai Jiao Tong University, 800 Dongchuan Road, Shanghai 200240, China; wangxinrui@sjtu.edu.cn (X.W.); 1131109036@alumni.sjtu.edu.cn (H.L.); xlwang@sjtu.edu.cn (X.W.); 2 Serionix, Inc. 60 Hazelwood Dr., Champaign, IL 61820, USA; wzheng@serionix.com; 3 Central Hospital of Fengxian District, South Hospital of the Sixth People’s Hospital, Shanghai 201499, China

**Keywords:** hybrid spheres, silver nanoparticle, *Lolium multiflorum*, toxicity

## Abstract

The rapid development of nanotechnology and its widespread use have given rise to serious concerns over the potential adverse impacts of nanomaterials on the Earth’s ecosystems. Among all the nanomaterials, silver nanoparticles (AgNPs) are one of the most extensively used nanomaterials due to their excellent antibacterial property. However, the toxic mechanism of AgNPs in nature is still unclear. One of the questions under debate is whether the toxicity is associated with the size of AgNPs or the silver ions released from AgNPs. In our previous study, a sub-micron hybrid sphere system with polydopamine-stabilized AgNPs (Ag@PDS) was synthesized through a facile and green method, exhibiting superior antibacterial properties. The current study aims to explore the unique toxicity profile of this hybrid sphere system by studying its effect on germination and early growth of *Lolium multiflorum*, with AgNO_3_ and 15 nm AgNPs as a comparison. The results showed the seed germination was insensitive/less sensitive to all three reagents; however, vegetative growth was more sensitive. Specifically, when the Ag concentration was lower than 40 mg/L, Ag@PDS almost had no adverse effects on the root and shoot growth of *Lolium multiflorum* seeds. By contrast, when treated with AgNO_3_ at a lower Ag concentration of 5 mg/L, the plant growth was inhibited significantly, and was reduced more in the case of AgNP treatment at the same Ag concentration. As the exposures of Ag@PDS, AgNO_3_, and AgNPs increased, so did the Ag content in the root and shoot. In general, Ag@PDS was proven to be a potential useful hybrid material that retains antibacterial property with light phytotoxicity.

## 1. Introduction

Nanoparticles (NPs) have attracted considerable interest due to their unique physicochemical properties compared with their bulk counterparts; thus, they are applied in various fields such as water treatment, cosmetics, medicine, food, and so on [1,2]. As a result, there is increasing environmental exposure to nanoparticles, which happens during the life cycle of NPs, such as manufacturing, transporting, atmospheric emission, effluent discharge, and agricultural use, etc. Despite the growing applications and widespread exposure to NPs, our knowledge of how they may affect natural ecosystems has not caught up. Increasing concerns are raised about the potential negative effects of NPs due to quantum size effects and their large specific surface area. Therefore, it is urgent to explore the potential impacts of nanomaterials on the environment and human health.

Over the past several decades, people not only care more about the toxicity and transportation of nanomaterials in the environment, but also have begun to focus on the probable health harms to living organisms [3,4,5]. The toxicity of nanoparticles (TiO_2_, ZnO, Ag, Cu, Al, carbon nanotubes, and so on) to living species has been studied in a wide range, including algae, higher plants, animals, and even humans [6,7,8,9,10]. For example, Atha et al. proposed that copper oxide nanoparticles strongly inhibited grassland plant growth and induced DNA damage as well as cyanobacteria, which are ancient prokaryotic microorganisms, by generating excess formation of reactive oxygen substances (ROS) [11].

Silver nanoparticles (AgNPs), with unique optical, electrical, thermal, and antibacterial properties, are one of the most widely used nanomaterials [12,13,14]. Their extensive uses may increase the chances of inadvertent release into the environment and ecosystem, of which plants hold a large percentage. NPs can reach plants through direct application, accidental release, contaminated soil/sediments, or atmospheric fallouts, which results in a significant negative effect on food crops and the food chain. It differs with the nanoparticles used, their size and concentration, and the plant species [15,16,17]. To date, there are limited studies that have reported on the impact of AgNPs on vascular plants; however, all of these studies suggested the detrimental effects of AgNPs on plant growth.

Some reports suggested that the biological effect of AgNPs could be observed at concentrations of even 1000 times lower than that for the dissolved Ag^+^ ions [18]. AgNPs have shown adverse effects on seed germinations, root, and shoot growth at concentrations of 4500 mg/L, 6000 mg/L, and 3000 mg/L on species of rice *(Oryza sativa*), Mung bean (*Vigna radiata*), and Chinese cabbage (*Brassica campestris*), respectively [19]. For *Cucurbita pepo*, AgNPs can induce several times more reductions in biomass and transpiration rates than bulk Ag [20]. Scherer et al. found that AgNPs had cytotoxic and genotoxic impacts on root cells of *Allium cepa* [21]. Krajcarová et al. characterized the spatial distribution of silver nanoparticles in root tissues of *Vicia faba* by laser-induced breakdown spectroscopy (LIBS) to check AgNPs’ toxicity to higher plants [22].

As for the virtue of AgNPs, although some effort has been made, it is still challenging to explore the possibilities of both keeping the antibacterial effort of AgNPs and reducing their side effects in the plant. In consideration of AgNPs’ toxicity, which can be attributed a lot to their small size, enlarging the AgNPs’ size while keep the resistance property to bacteria may be an effective solution [23]. On the other hand, polydopamine has good biocompatibility and excellent biodegradability [24], which is the essential component of natural melanin named eumelanin [25]. Due to many functional groups it has, such as catechol and amine, polydopamine has excellent chemical reactivity to build diverse hybrid materials by multi-size spheres [26]. Based on these advantages above and our group’s experiences in polydopamine [27], in our previous study, size-controllable hybrid spheres of silver nanoparticles loaded on sub-micrometer polydopamine spheres (Ag@PDS) were fabricated by a facile and green method with good antibacterial property and theyexhibited a good biocompatibility [28]. The current study attempted to investigate the effects of the kind of silver nanoparticle hybrid spheres Ag@PDS on plant germination and early growth, and we exposed seeds of the annual ryegrass *L. multiflorum* to a range of exposure concentrations of Ag@PDS, AgNPs, and Ag ions (AgNO_3_). The research helps to understand the toxicity mechanism of larger size Ag-containing particles and their influences on the *L. multiflorum*. Furthermore, it also provides some support that the Ag@PDS could be a perfect solution not only to reduce the adverse impact of AgNPs, but also to offer a better antibacterial effect for more plants.

## 2. Materials and Methods

### 2.1. Materials

Dopamine hydrochloride (99%+, Aladdin reagent, Shanghai, China) was directly used without any further treatment. Potassium nitrate (99.5%), silver nitrate (99.8%), ethanol (99.7%), and ammonia aqueous solution (25–28 wt.%, mixture) were bought from Sinopharm Chemical Reagent Co., Ltd. (Shanghai, China). *L. multiflorum* seeds were obtained from Suzhou Oushang Gardening Company (Suzhou, China). Seeds were first sterilized by soaking them with 4% NaClO solution for 10 min and thoroughly cleaned with deionized water to avoid surface adherents. Pure AgNPs were synthesized according to the previous study [29]. AgNPs were uniform with the mean diameter of about 15 nm, whose size was similar to those AgNPs attached to the surface of PDS microspheres in Ag@PDS.

### 2.2. Synthesis of Polydopamine Microsphere-Stabilized Silver Nanoparticles (Ag@PDS)

The synthesis of Ag@PDS was based on our previous study [28]. First, 2 mL ammonia aqueous solution (NH_4_OH, 25%) was added slowly into the mixture of 90 mL deionized water and 40 mL ethanol under a stirring condition, and then homogenized for 30 min at 25 °C. The dopamine hydrochloride aqueous solution (0.5 g dopamine hydrochloride dissolved in 10 mL deionized water) was injected into the above mixture solution, stirring for 30 h. After centrifugation and rinsing with water for 3 cycles, the polydopamine spheres (PDS) were finally prepared. The size of the PDS was well controlled by changing the amount of NH_4_OH [30].

The as-prepared PDS was fully dispersed in deionized water via agitation and sonication for several minutes. AgNO_3_ aqueous solution (0.8 g silver nitrate dissolved in 10 mL deionized water) was added into the PDS dispersion dropwise in an iced bath under sonication conditions, and the reduction lasted for 10 min. After centrifugation and rinsing with water for 3 cycles, the Ag@PDS products, drying at 60 °C in a vacuum, were prepared for further application.

### 2.3. Characterization of Polydopamine Sphere-Stabilized Silver Nanoparticles (Ag@PDS)

Transmission electron microscopy (TEM) was used to characterize the morphology of samples under 100 kV accelerating voltage, which was equipped with an Oxford INCA Energy TEM 200 EDX system (JEM-2100, JEOL, Tokyo, Japan). Dynamic light scattering (DLS) was used to investigate particle size distribution (Zetasizer ZS, Malvern Panalytical, Malvern, UK). The chemical composition of PDS and Ag@PDS was investigated by the Fourier transform infrared spectroscopy (Perkin-Elmer, Waltham, MA, USA). The X-ray diffraction (XRD) of Ag@PDS was measured by Rigaku D/Max 2550 (Rigaku, Tokyo, Japan), and the 2θ scanning was set in the range of 5°–90° (Cu Kα, λ = 1.5418Å). Germination patterns of the *L. multiflorum* roots were observed by microscopic photos (Zeiss Lumar V12 stereoscope, Carl Zeiss AG, Jena, Thuringia, Germany). After digestion with 5% HNO_3_ for at least 24 h, the silver concentration of Ag@PDS was measured by an atomic absorption spectrophotometer (Perkin-Elmer, Waltham, MA, USA).

### 2.4. Plant Germination and Early Growth in Hydroponic Culture

The toxicity of each independent nanoparticle aqueous suspension was evaluated by exposing seeds of *L. multiflorum* to six different silver concentrations (5, 10, 20, 40, 60, and 80 mg/L) of AgNPs, Ag@PDS, AgNO_3_, and control groups (DI water, 23.8 mg/L KNO_3_ equivalents to NO_3_^−^ in 40 mg/L Ag content of AgNO_3_, and 960 mg/L PDS equivalent to PDS in 40 mg/L Ag content of Ag@PDS).

In each individual treatment group, about 200 *L. multiflorum* seeds were soaked in 5 mL of test sample for 1 h, and each group was shaken during the hour for 5 s for three cycles to assure all the seeds were fully mixed with the suspensions. A piece of filter paper was added into each 100 mm × 15 mm petri dish, and 4 mL of the test sample (solution or suspension) was immediately added after sonication. Then, 35 seeds were shifted onto the filter paper equidistantly. Petri dishes were then covered and sealed with parafilm and put evenly in the greenhouse. The temperature condition was set in the range of 25–30 °C for 16 h (daylight) and 15–20 °C for 8 h (night).

Five repeated samples were performed for each test sample (nanoparticle suspension). The germination rate of the seed, length, and biomass of both the shoot and root were measured and recorded after incubation for 7 days. The germination rate was determined via the method that comparing the number of seeds developing one at least 3 mm simple primary root (half the mean size of the seed) with the total number of seeds in each separated petri dish.

### 2.5. Silver Content in Dry Plant Tissue

*L. multiflorum* seedlings exposed to treatment suspensions were harvested after 7 days of cultivation to avoid contamination from the growth matrix carefully. The root and shoot were separated and cleaned with deionized water to avoid adherent, and then totally dried in a hot-air oven, followed by HNO_3_ digestion until a colorless 1 mL solution/suspension was retained. The prepared solutions were filtered with 0.45 μm filter membrane and added up to 25 mL with deionized water. Silver concentrations in aqueous samples of digested *L. multiflorum* seedlings were also characterized via AAS.

### 2.6. Microscopy

A comparative analysis of fresh roots growing in the AgNPs, Ag@PDS, AgNO_3_, and control treatments was conducted by using light microscopy (LM) to investigate the cell division state and morphology of the root tips. Roots of all treatments were evenly cleaned with deionized water. We checked 1 cm above the primary root tip for each individual seedling.

### 2.7. Statistical Analysis

Each treatment was repeated three times in multiple experiments, and the total arithmetical mean length or mass of the simple primary root and shoot was recorded after growing for 7 days. We applied one-way ANOVA to calculate variations between treatments for the individual plant responses. The HSD was used to check the statistical differences. Data were the output as the mean ± standard deviation (SD), and *p* ≤ 0.05 was deemed to be significantly different.

## 3. Results

### 3.1. Characterization of Ag@PDS

**Figure 1:**
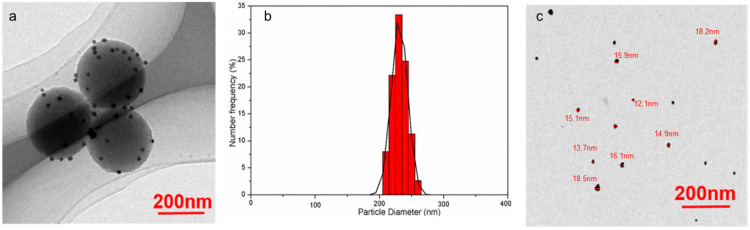
TEM images (**a**) and DLS curve (**b**) of the polydopamine sphere embellished with silver nanoparticles (Ag@PDS), and TEM images (**c**) of AgNPs.

TEM images of Ag@PDS (Figure 1a and Supplementary Materials, Figure S5) illustrate that AgNPs were attached onto the surface of PDS uniformly, and the diameter of Ag@PDS was 230 nm. The energy dispersive X-ray spectrum (EDX) and XRD patterns of obtained Ag@PDS (Supplementary Materials, Figure S1) can also prove the existence of AgNPs on the surface of PDS. The attached Ag nanoparticles on the Ag@PDS were spherical, with a particle size around 12–18 nm. This sphere structure could effectively prevent the aggregation of silver nanoparticles. The dynamic light scattering (DLS) results (Figure 1b) showed that Ag@PDS was about 230 nm with a narrow size distribution, which is consistent with the size measured by TEM.

Besides, the Ag@PDS hybrid sphere can disperse well and remain stable in water and other common solvents. The Ag@PDS was ultrasonicated in deionized water for 30 min before TEM scanning. As the TEM image (Figure 1a) shows, after ultrasonication, AgNPs were still attached on the surface of the polydopamine sphere and no stand-alone AgNPs were found. In addition, the silver ion released from the Ag@PDS was barely measurable (<5 ppm) during the span of the 7-day observation time (SI, Figure S2). Such results prove that the bonding between AgNPs and PDS is strong and stable, making sure that the nanostructure does not prohibit the application of Ag@PDS in various fields with concerns of leaching. Antibacterial properties of Ag@PDS were studied by our previous work [28]. Data on the bactericidal properties of AgNPs-PDS with the Gram-negative bacteria *E. coli* and Gram-positive *S. aureus* can be found in the Supplementary Materials (Figure S6).

### 3.2. Effects of Ag@PDS, AgNO_3_, and AgNPs on Seed Germination

**Figure 2:**
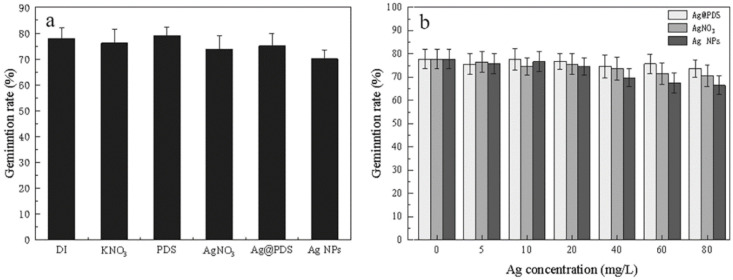
Germination rate of *L. multiflorum* seeds in control and Ag@PDS, AgNO_3_, and AgNP treatments. Equivalent control condition of DI, KNO_3_, PDS, and 40 mg/L Ag concentration of Ag@PDS, AgNO_3_, and AgNPs (**a**); different Ag concentrations of Ag@PDS, AgNO_3_, and AgNPs (*p* ≤ 0.05) (**b**).

Seed germination was studied in different suspensions of Ag@PDS and AgNPs and in the solution of Ag^+^ in comparison with control groups. As shown in Figure 2a, there were almost no statistically significant differences in *L. multiflorum* seed germination among three reagents and control groups at the same concentration of 40 mg/L. As shown in Figure 2b, with increasing concentrations, the germination patterns of the seeds treated with Ag@PDS at various Ag concentrations exhibited small differences, which are close to those of control groups in Figure 2a. However, at the Ag concentration of 80 mg/L, AgNPs and Ag^+^ exhibited inhibitory effect on seed germination, down to 67% for AgNPs and 71% for Ag^+^. In comparison, Ag@PDS at all the concentrations tested (5–80 mg/L) had a minimal/unnoticeable impact on the germination rate.

The unaffected *L. multiflorum* seed germination could be explained by seed coating protection mechanism, which impeded interactions between the silver and seeds [31]. The above experimental results proved that silver nanoparticles can protect seeds against fungi, treating seeds with either Ag@PDS or low-concentration AgNPs did not impede germination. Therefore, it is possible to apply this treatment in agricultural practices to reduce the environmental impacts of traditional fungicides. As suggested, Ag@PDS or AgNPs can protect seeds against fungi, which implies the possibility of agricultural practices to use AgNPs as fungicides for seed treatment with minimum impact on seed germination.

### 3.3. Effects of Ag@PDS, AgNO_3_, and AgNPs on Root and Shoot Elongation

Exposure time over 15-day periods of different reagents was carried out. Due to the results (Supplementary Materials, Figure S3), we chose 7 days as the optimum exposure time.

**Figure 3:**
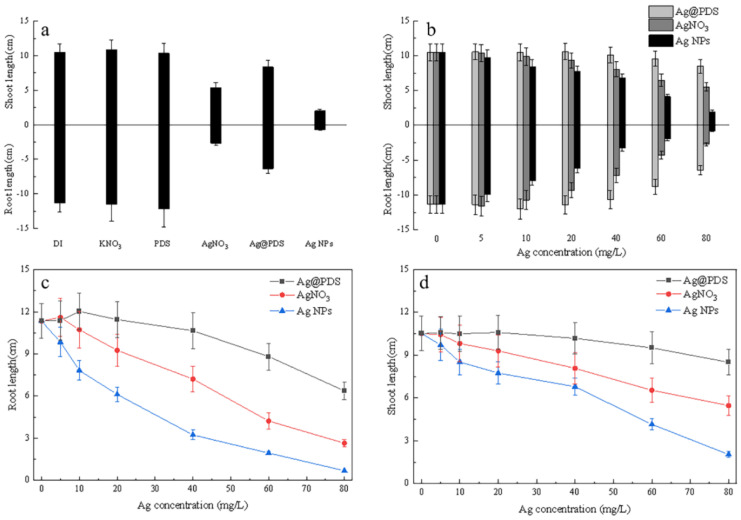
Effect of Ag@PDS, AgNO_3_, and AgNPs on the *L. multiflorum* root (**a**,**c**) and shoot (**b**,**d**) length after 7 days of exposure (*p* ≤ 0.05).

As the seedlings grew, silver treatments on the *L. multiflorum* seeds showed significant differences (Figure 3). The shoot and root growth were observed to be more sensitive to Ag exposure treatment than previously reported seed germination [20]. Probably because of more exposure to silver, the roots showed toxic symptoms more prominently than the shoots.

The AgNPs and AgNO_3_ had a significant inhibitory effect on the root and shoot length in a dose-dependent manner even at low concentrations, and at the same concentrations AgNPs had a stronger inhibitory effect than AgNO_3_, the maximum reduction achieved at 80 mg/L was 93.4% and 76.4%, respectively. While the root and shoot elongation patterns of the *L. multiflorum* seeds treated with Ag@PDS (silver element was characterized by concentrations lower than 20 mg/L in the system) did not change compared to the control group. With the concentration increasing, the Ag@PDS started to have a noticeable inhibition on root and shoot length, but only the highest concentration of Ag@PDS (Ag concentration 80 mg/L) induced a considerable restraint of root elongation and shortened the root length to 56.1% after 7 days of exposure.

In general, for all three cases, Ag showed a dose-dependent inhibitory effect on both the root and shoot length. At the same concentration, the inhibitory effect increased in the order of Ag@PDS, Ag ions, and AgNPs. For root length, Ag@PDS showed no adverse impact at <40 mg/L, while Ag ions and AgNPs exhibited an 18.4% and 46.0% reduction at 20 mg/L, respectively. At 40 mg/L, Ag@PDS reduced root length slightly to 93.7%, compared with 63.7% and 32.2% for Ag ions and AgNPs, respectively. This implies that Ag@PDS < 40 mg/L could be a good choice, while AgNPs and Ag ions > 80 mg/L exhibit detrimental effects on root growth.

For shoot length, the same effect applies, while to slightly less extent, especially for Ag@PDS, which only showed obvious differences at concentrations > 60 mg/L.

### 3.4. Effects of Ag@PDS, AgNO_3_, and AgNPs on Root and Shoot Biomass

Correspondingly, the root and shoot weight of *L. multiflorum* showed a similar trend as the root and shoot elongation; roots appeared more sensitive to Ag treatment than the shoots from their first contact of the toxicant, and no significant inhibitive effects were observed at the low concentration (40 mg/L) of Ag@PDS (Supplementary Materials, Figure S4). When the concentrations of Ag@PDS were lower than 40 mg/L, the dry biomass of roots was not affected, which is the same case as exposure to 5, 10, and 20 mg/L Ag@PDS. In comparison, AgNO_3_ and AgNPs had a significant inhibition on the root and shoot biomass in a dose-dependent manner from 5 mg/L. It was also noticeably decreased by Ag treatment with the high concentration. Exposure to Ag@PDS at 40 and 80 mg/L reduced *L. multiflorum* root biomass by 6.3% and 43.9%, respectively. After exposure to AgNO_3_ and AgNPs for one week, the dry biomass of the roots was reduced to 62.1% and 32.1% at 40 mg/L and only 27.9% and 9.4% at 80 mg/L of the control, respectively. Thus, the inhibition threshold and degree of Ag treatment on *L. multiflorum* are both different in germinating period and seedling period, suggesting that the sensitivity to the toxicant of a plant significantly depends on its growth period.

### 3.5. Effects of Ag@PDS, AgNO_3_ and AgNPs on Ag Uptake

**Figure 4:**
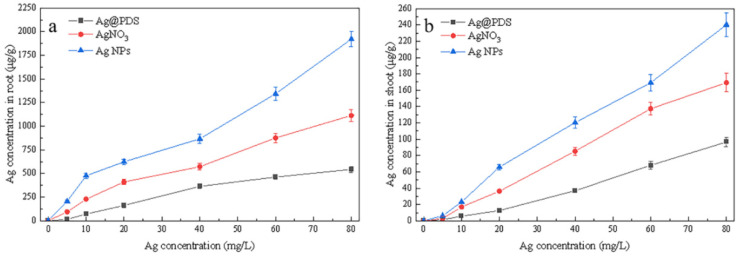
Ag content in the *L. multiflorum* root (**a**) and shoot (**b**) after 7 days exposure of Ag@PDS, AgNO_3_, and AgNPs.

After 7 days of exposure to the Ag@PDS suspension, AgNO_3_ solution, and AgNP suspension, the Ag contents in *L. multiflorum* tissues were characterized by atomic absorption spectroscopy, as shown in Figure 4. With the concentrations of Ag in the suspensions or solution increasing, the Ag content exhibited an increasing trend both in the root and shoot of the treated plant, with a total uptake of up to 2000 μg/g Ag content.

As shown in Figure 4a, with the Ag concentration rising, a notable increase of Ag^+^ content in roots was observed when treated with AgNO_3_ and AgNPs, reaching 102.3 μg/g and 213.8 μg/g, respectively, which are 3.6 times and 8.6 times higher than that of the roots treated with Ag@PDS (22.2 μg/g), respectively. The Ag^+^ uptake in the *L. multiflorum* tissues originated from the released Ag^+^ by AgNO_3_, AgNPs, and Ag@PDS. Only a few Ag^+^ released from Ag@PDS because of the strong bonding between AgNPs and PDS, so the Ag content in the roots treated with Ag@PDS was at the lowest level. In comparison, there was a large amount of Ag^+^ in AgNO_3_ solution, facilitating the Ag^+^ absorption of the root.

As shown in Figure 4b, at the concentration of 80 mg/L, the Ag content reached a maximum value of 240.2 µg/g in the AgNP-treated shoot, higher than those in the shoots of AgNO_3_- and Ag@PDS-treated plants, which accumulated only 169.5 µg/g and 96.8 µg/g, respectively. Such a phenomenon is accountable when considering that, upon the contact of AgNPs and cellular membranes, the formation of reactive oxygen species was induced and the damage to the cellular membranes was caused subsequently, facilitating the adsorption and internalization of the Ag content [32].

In addition, the Ag content uptake range of 0–2000 μg/g in the root was much broader than that of 0–240 μg/g in the shoot. Even at the same concentration of Ag, the Ag content uptake in the root was always much higher than that in the shoot. Such a phenomenon indicates that the majority of Ag content aggregates in the root part and the minority in the shoot part. Translocation factors (TFs) of Ag, defined as the Ag content ratio of the shoot to root, were very low in *L. multiflorum*, probably ascribed to that the majority of AgNPs adsorbed on the root surface, and only a little Ag^+^ could enter the vessel for further transport [33].

### 3.6. Effects of Ag@PDS, AgNO_3_, and AgNPs on Root Morphology

**Figure 5:**
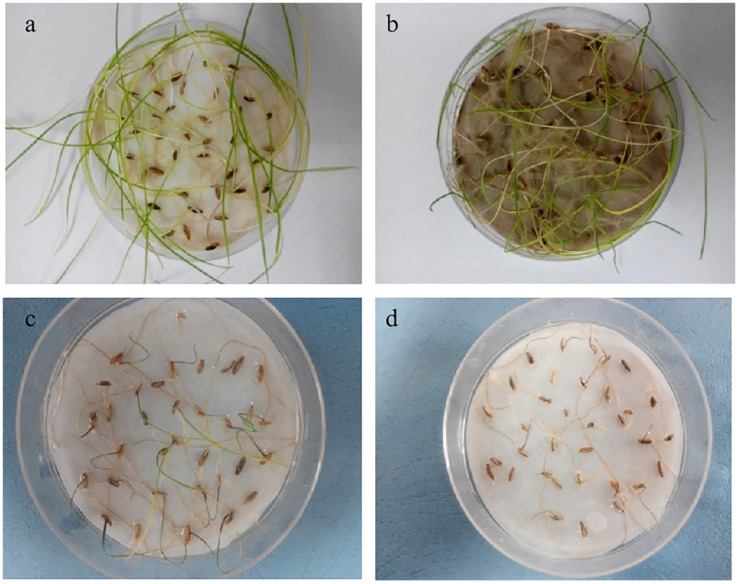
Photo of *L. multiflorum* growth. DI (**a**); 40 mg/L Ag content Ag@PDS (**b**); 40 mg/L Ag content AgNO_3_ (**c**); 40 mg/L Ag content AgNPs (**d**).

Plants treated with AgNO_3_ at Ag content of 40 mg/L were thinner in roots than the control group and Ag@PDS-treated group, and the AgNP-treated group showed the thinnest roots (Figure 5). In addition, Ag@PDS-treated shoots expressed only a little brown in the shoot tip, while more brown areas were observed in the shoots treated with AgNO_3_, and almost full dark brown areas all over the shoot were seen in the AgNP-treated shoots.

The “brown spots” on shoots were found to be associated with the adverse result of Ag. The excess Ag exhibited some oxidative stress towards the *L. multiflorum* tissue, impeding the photosynthesis process, thus decreasing metabolic process rates and the level of chlorophyll [32]. Such “brown spots” are a quantifiable and repeatable dose-dependent phenomenon, even though it has still not been completely determined whether the brown spots were induced by the dissolved Ag^+^ or the complexes consisting of Ag^0^ and plant secondary compounds such as tannins or anthocyanins.

**Figure 6:**
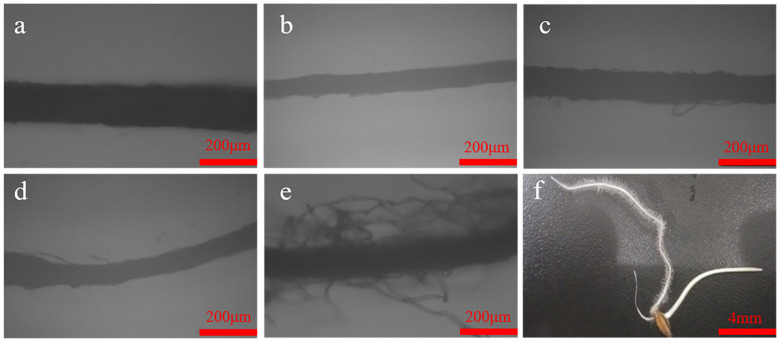
Light microscopic observation of *L. multiflorum* root hairs after 7 days exposure of DI (**a**); 23.8 mg/L KNO_3_ (**b**); 40 mg/L Ag content of AgNO_3_ (**c**); 40 mg/L Ag content of AgNPs (**d**); 40 mg/L Ag content of Ag@PDS (**e**); photo of fibrous root of *L. multiflorum* in 40 mg/L Ag content of Ag@PDS (**f**).

As the light microscopic observations of the seedlings revealed in Figure 6, the PDS- and Ag@PDS-treated roots developed long root hairs, while no root hairs were observed in others including the control DI, and the KNO_3_-, AgNO_3_- and AgNP-treated roots. Such a phenomenon indicates that the introduction of PDS may help to develop root hairs. It can be concluded that the combination of AgNPs and PDS was good, as it not only remains antibacterial, but also helps to develop root hairs, enhancing roots stability to the earth and promoting the absorption of water and nutrients.

## 4. Discussion

Silver nanoparticles (AgNPs) have unique properties due to the size effects and large surface area, but at the same time, they may induce toxicity to organisms. It is important to keep the good antibacterial property of AgNPs, while reducing their toxicity on creatures. In this work, a sub-micro hybrid sphere system with polydopamine-stabilized silver nanoparticles, which have shown superior antibacterial properties (Supplementary Materials, Figure S6), was investigated on its effects on the growth of the *L. multiflorum*.

In the literature, it is known that studying seed germination and root early growth is a widely used method to test the acute phytotoxicity of nanoparticles [31]. For this test, the toxicity of nanoparticles can be typically attributed to two different actions: (1) chemical toxicity based on chemical composition (e.g., the release of toxic ions) or (2) physical toxicity (e.g., stress or stimuli caused by the surface, size, and shape of the particles). For a specific AgNPs system, the phytotoxicity depends on various factors including the plant species and the AgNPs’ size, concentration, exposure condition, and adsorption material morphologies, etc.

It has been reported that a smaller size of AgNPs introduces greater toxicity into the plant. An example is that about 1.4 nm Ag colloid has greater toxicity than AgNPs of 5 and 20 nm on flax, barley, and ryegrass [34]. On the other hand, the presence of other chemical components in AgNPs has also significantly affected seed germination rates for multiple plant species [35].

The present study revealed that the tested concentration of Ag@PDS, AgNO_3_, and AgNPs did not affect the seed germination rate of the *L. multiflorum* plant because of the protection of the seeds’ thick coat. Similar effects were also found in the cases of other plants [36]. *L. multiflorum* was more sensitive to AgNPs and AgNO_3_ at the vegetative growth stage than the germination stage. At the vegetative growth stage, Ag@PDS almost had no adverse effect on the plant growth at a low concentration, while AgNO_3_ inhibited the growth slightly, and seeds exposed to AgNPs showed severely inhibited growth of roots and shoots. The discovery of such phenomena demonstrates that the toxicity of AgNPs exceeded that of identical doses of dissolved Ag (such as AgNO_3_), indicating that AgNPs toxicity could be mainly directly attributed to the silver nanoparticles themselves. Because of its larger size, Ag@PDS indeed reduced the toxicity of the AgNPs to a large extent.

In terms of the toxic mechanism of AgNPs in various living organisms, it is generally agreed that the toxicity of silver nanoparticles is mainly attributed to the presence of the nanoparticles, partly related to the released Ag^+^ ions from the nanoparticles or the free radicals generated in the AgNP hybrids [37,38].

Similar to these previous findings, AgNPs caused more inhibition of the growth of the roots than the shoots of *L. multiflorum*. This is because silver (Ag^+^ or other forms) was firstly accumulated in the roots and subsequently translocated to the shoots after the exposure to AgNPs, which was proved by the results of AAS. This was another example for the accumulation and translocation sequence of AgNPs: border cells, root cap, then columella, and finally columella initials [33]. Meanwhile, the vacuoles and cell walls in the root cells of plant samples broke up due to the toxicity caused by the AgNPs, and then the nanoparticles were absorbed and translocated from the roots to the leaves. As a result, AgNPs can kill the plant root cells and interfere with intracellular components, inhibiting the plants’ growth.

In this work, the inhibition of plant growth by Ag-containing chemicals and high Ag ion concentrations was caused through the uptake, translocation, and bioaccumulation of Ag in *L. multiflorum* shoots. Furthermore, the translocation factor of Ag in the *L. multiflorum* treated with Ag@PDS, AgNO_3_, and AgNPs showed that the movement of Ag from roots to shoots in these situations was different. Currently, we guess that one possible explanation is that, because of the firm and stable bonding between AgNPs and PDS, the direct transport of AgNPs was difficult, and the transport of Ag ions released from the Ag@PDS was followed by a reduction to the elemental form in the plant constituting the main Ag accumulation. The roots and shoots of *L. multiflorum* in the Ag@PDS suspension contained little Ag chemicals compared to those in the AgNPs and in the AgNO_3_ treatment, mainly because the silver ions released from Ag@PDS were too little to produce enough Ag deposits in the roots or shoots. Therefore, it is concluded that increased ion dissolution from the nanoparticles only partly explains the observed toxicity to *L. multiflorum*. A more important factor is the size of the Ag nanoparticles. Compared with AgNO_3_, AgNPs showed stronger toxicity to *L. multiflorum*, indicating that growth inhibition and cell damage of plants can not only be attributed to the dissolved silver ions released from the AgNPs, but also can be directly attributed to the nanoparticles themselves, and the latter seems to play a more important role in AgNPs toxicity.

## 5. Conclusions

Nanosized silver possesses excellent antibacterial properties, but it also causes both environmental and health concerns. It is a challenge to keep its superior antibacterial properties without introducing toxicity. The effects of the hybrid spheres were studied on germination and early growth of *L. multiflorum* system. Our results are in agreement that synthesized high antimicrobial Ag@PDS have little effect on seed germination or root and shoot early growth in *L. multiflorum*, and Ag@PDS may even have a favorable effect on *L. multiflorum* as they help to develop lots of root hairs to enhance roots’ stability to the earth and promote the absorption of water and nutrients. We can conclude that Ag@PDS have less toxicity to *L. multiflorum* seeds in comparison with AgNPs or AgNO_3_ and may be used for agriculture with a tested profile at permissible levels (lower than Ag content of 40 mg/L). The results of this study can also provide a path for how to design a hybrid nanoparticle system with less toxicity. This may open a pathway of utilizing nanoparticles more safely without jeopardizing their antibacterial performance for various other applications as well.

## Data Availability

Data, associated metadata, and calculation tools are available from the corresponding author (zzheng@sjtu.edu.cn).

## Supplementary Materials

The following are available online at https://www.mdpi.com/article/10.3390/toxics9070151/s1, Figure S1: The energy dispersive X-ray spectrum (EDX) confirms the existence of Ag in Ag@PDS (**a**). The X-ray diffraction (XRD) patterns of the obtained Ag@PDS (**b**) evidently reveal that silver ions have been reduced to 0 value of metallic. FTIR spectra of polydopamine spheres (PDS) and polydopamine spheres loaded with silver nanoparticles (Ag@PDS) (**c**). Figure S2. The silver ions released from the Ag@PDS and 15nm AgNPs are barely measurable (<5 ppm) during the observation time with the same Ag concentration. Figure S3. Root length of *Lolium multiflorum* after several days of exposure of 40mg/L of Ag@PDS, AgNO_3_, and AgNPs. Figure S4. Effect of DI, KNO_3_, PDS, AgNO_3_, Ag@PDS, and AgNPs on the *Lolium multiflorum* length (**a**) and mass (**b**) after 7 days of exposure. Figure S4. Effect of Ag@PDS, AgNO_3_, and AgNPs on the *Lolium multiflorum* root (**a**,**c**) and shoot (**b**,**d**) mass after 7 days of exposure (*p* ≤ 0.05). Figure S5. TEM images of polydopamine spheres loaded with silver nanoparticles (Ag@PDS) with 50 nm scale bar. Figure S6. Images of LB-agar plates, which were used in the antibacterial activity experiment of samples against *E.coli*: control (**a**), polydopamine (PDS) (**b**), polydopamine sphere loaded with silver nanoparticles (Ag@PDS) (**c**).

## References

[1] Chen G., Roy I., Yang C., Prasad P.N. (2016). Nanochemistry and Nanomedicine for Nanoparticle-based Diagnostics and Therapy. Chem. Rev..

[2] Perreault F., De Faria A.F., Elimelech M. (2015). Environmental applications of graphene-based nanomaterials. Chem. Soc. Rev..

[3] Louie S.M., Tilton R.D., Lowry G.V. (2016). Critical review: Impacts of macromolecular coatings on critical physicochemical processes controlling environmental fate of nanomaterials. Environ. Sci. Nano.

[4] Garner K.L., Suh S., Lenihan H.S., Keller A.A. (2015). Species Sensitivity Distributions for Engineered Nanomaterials. Environ. Sci. Technol..

[5] Piperigkoua Z., Karamanoua K., Engind A.B., Gialeli Ch Docea A.O., Vynios D.H. (2016). Emerging aspects of nanotoxicology in health and disease: From agriculture and food sector to cancer therapeutics. Food Chem. Toxicol..

[6] Schwab F., Zhai G., Kern M., Turner A., Schnoor J.L., Wiesner M.R. (2016). Barriers, pathways and processes for uptake, translocation and accumulation of nanomaterials in plants—Critical review. Nanotoxicology.

[7] Ribeiro F., Van Gestel C.A., Pavlaki M.D., Azevedo S., Soares A.M., Loureiro S. (2017). Bioaccumulation of silver in *Daphnia magna*: Waterborne and dietary exposure to nanoparticles and dissolved silver. Sci. Total. Environ..

[8] Shaw B.J., Liddle C.C., Windeatt K.M., Handy R.D. (2016). A critical evaluation of the fish early-life stage toxicity test for engineered nanomaterials: Experimental modifications and recommendations. Arch. Toxicol..

[9] Olasagasti M., Gatti A.M., Capitani F., Barranco A., Pardo M.A., Escuredo K., Rainieri S. (2014). Toxic effects of colloidal nanosilver in zebrafish embryos. J. Appl. Toxicol..

[10] Gatti A.M., Montanari S., Ferrero S., Lavezzi A.M. (2021). Silver nanoparticles in the fetal brain: New perspectives in understanding the pathogenesis of unexplained stillbirths. Nanomedicine.

[11] Atha D.H., Wang H., Petersen E.J., Cleveland D., Holbrook R.D., Jaruga P., Dizdaroglu M., Xing B., Nelson B.C. (2012). Copper Oxide Nanoparticle Mediated DNA Damage in Terrestrial Plant Models. Environ. Sci. Technol..

[12] Singh S., Bharti A., Meena V.K. (2015). Green synthesis of multi-shaped silver nanoparticles: Optical, morphological and antibacterial properties. J. Mater. Sci. Mater. Electron..

[13] Peng P., Hu A., Gerlich A.P., Zou G., Liu L., Zhou Y.N. (2015). Joining of Silver Nanomaterials at Low Temperatures: Processes, Properties, and Applications. ACS Appl. Mater. Interfaces.

[14] Li R.-Z., Hu A., Bridges D., Zhang T., Oakes K.D., Peng R., Tumuluri U., Wu Z., Feng Z. (2015). Robust Ag nanoplate ink for flexible electronics packaging. Nanoscale.

[15] Jiang H.S., Li M., Chang F.-Y., Li W., Yin L.-Y. (2012). Physiological analysis of silver nanoparticles and AgNO3 toxicity to *Spirodela polyrhiza*. Environ. Toxicol. Chem..

[16] Giuseppe C., Anna P. (2015). Penetration and toxicity of nanomaterials in higher plants. Nanomaterials.

[17] Devi M.S., AshokKumar K., Annapoorani S. (2017). Phytofabrication and encapsulated of silver nanoparticles from *Gloriosa superba*. Mater. Lett..

[18] Lee D.-Y., Fortin C., Campbell P.G. (2005). Contrasting effects of chloride on the toxicity of silver to two green algae, *Pseudokirchneriella subcapitata* and *Chlamydomonas reinhardtii*. Aquat. Toxicol..

[19] Aslani F., Bagheri S., Muhd Julkapli N., Juraimi A.S., Hashemi F.S.G., Baghdadi A. (2014). Effects of Engineered Nanomaterials on Plants Growth: An Overview. Sci. World J..

[20] Musante C., White J.C. (2012). Toxicity of silver and copper to *Cucurbita pepo*: Differential effects of nano and bulk-size particles. Environ. Toxicol..

[21] Scherer M.D., Sposito J.C., Falco W.F., Grisolia A.B., Andrade L.H., Lima S.M., Machado G., Nascimento V.A., Gonçalves D.A., Wender H. (2019). Cytotoxic and genotoxic effects of silver nanoparticles on meristematic cells of *Allium cepa* roots: A close analysis of particle size dependence. Sci. Total. Environ..

[22] Krajcarová L., Novotný K., Kummerová M., Dubová J., Gloser V., Kaiser J. (2017). Mapping of the spatial distribution of silver nanoparticles in root tissues of *Vicia faba* by laser-induced breakdown spectroscopy (LIBS). Talanta.

[23] Yin L., Cheng Y., Espinasse B., Colman B.P., Auffan M., Wiesner M., Rose J., Liu J., Bernhardt E.S. (2011). More than the ions: The effects of silver nanoparticles on *Lolium multiflorum*. Environ. Sci. Technol..

[24] Liu Y., Ai K., Lu L. (2014). Polydopamine and its derivative materials: Synthesis and promising applications in energy, environmental, and biomedical fields. Chem. Rev..

[25] Simon J.D., Peles D.N. (2010). The red and the black. Acc. Chem. Res..

[26] Meng H., Mi B. (2013). Enabling graphene oxide nanosheets as water separation membranes. Environ. Sci. Technol..

[27] Dai F., Chen F., Wang T., Feng S., Hu C., Wang X., Zheng Z. (2016). Effects of dopamine-containing curing agents on the water resistance of epoxy adhesives. J. Mater. Sci..

[28] Luo H., Gu C., Zheng W., Dai F., Wang X., Zheng Z. (2015). Facile synthesis of novel size-controlled antibacterial hybrid spheres using silver nanoparticles loaded with polydopamine spheres. RSC Adv..

[29] Cui J., Hu C., Yang Y., Wu Y., Yang L., Wang Y., Liu Y., Jiang Z. (2012). Facile fabrication of carbonaceous nanospheres loaded with silver nanoparticles as antibacterial materials. J. Mater. Chem..

[30] Ai K., Liu Y., Ruan C., Lu L., Lu G. (2013). (Max) Sp2C-Dominant N-Doped Carbon Sub-micrometer Spheres with a Tunable Size: A Versatile Platform for Highly Efficient Oxygen-Reduction Catalysts. Adv. Mater..

[31] Parveen A., Rao S. (2014). Effect of Nanosilver on Seed Germination and Seedling Growth in *Pennisetum glaucum*. J. Clust. Sci..

[32] Qian H., Peng X., Han X., Ren J., Sun L., Fu Z. (2013). Comparison of the toxicity of silver nanoparticles and silver ions on the growth of terrestrial plant model *Arabidopsis thaliana*. J. Environ. Sci..

[33] Cui D., Zhang P., Ma Y.-H., He X., Li Y.-Y., Zhao Y.-C., Zhang Z.-Y. (2014). Phytotoxicity of silver nanoparticles to cucumber (*Cucumis sativus*) and wheat (*Triticum aestivum*). J. Zhejiang Univ. A.

[34] El-Temsah Y.S., Joner E.J. (2010). Impact of Fe and Ag nanoparticles on seed germination and differences in bioavailability during exposure in aqueous suspension and soil. Environ. Toxicol..

[35] Yin L., Colman B.P., McGill B.M., Wright J.P., Bernhardt E.S. (2012). Effects of Silver Nanoparticle Exposure on Germination and Early Growth of Eleven Wetland Plants. PLoS ONE.

[36] Yasur J., Rani P.U. (2013). Environmental effects of nanosilver: Impact on castor seed germination, seedling growth, and plant physiology. Environ. Sci. Pollut. Res..

[37] Liu J., Hurt R.H. (2010). Ion release kinetics and particle persistence in aqueous nano-silver colloids. Environ. Sci. Technol..

[38] Zhao C.-M., Wang W.-X. (2012). Importance of surface coatings and soluble silver in silver nanoparticles toxicity to *Daphnia magna*. Nanotoxicology.

