# Supplementary material for: Effects of Polydopamine Microspheres Loaded with Silver Nanoparticles on Lolium multiflorum: Bigger Size, Less Toxic
Source: Toxics. 2021 Jun 29;9(7):151. doi: 10.3390/toxics9070151 (PMC8309745; doi:10.3390/toxics9070151)
Supplement: Supplementary file 1 [file toxics-09-00151-s001.zip › toxics-1189118-supplementary.pdf]

Supplementary Materials: Effects of Polydopamine Microspheres Loaded with Silver Nanoparticles on *Lolium multiflorum*: Bigger Size, Less Toxic

Xinrui Wang, Hongyong Luo, Weihua Zheng, Xinling Wang, Haijun Xiao and Zhen Zheng

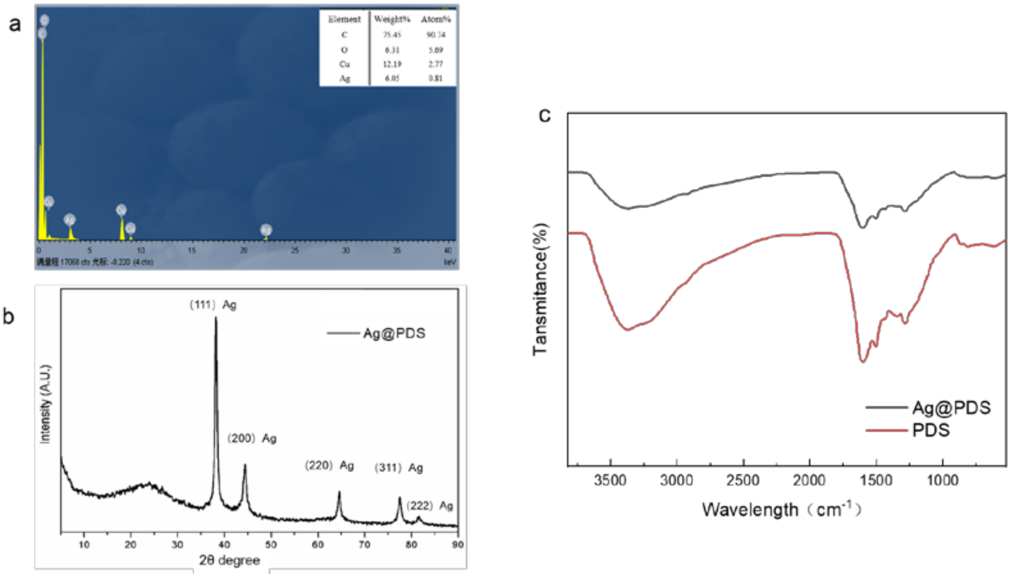

Figure S1. The energy dispersive X-ray spectrum (EDX) confirms well the existence of Ag in Ag@PDS (**a**). The X-ray diffraction (XRD) patterns of the obtained Ag@PDS (**b**) evidently reveal that silver ions have been reduced to 0 value of metallic.(**c**) FT-IR spectra of poly-dopamine spheres (PDS) and poly-dopamine spheres loaded with silver nanoparticles (Ag@PDS).

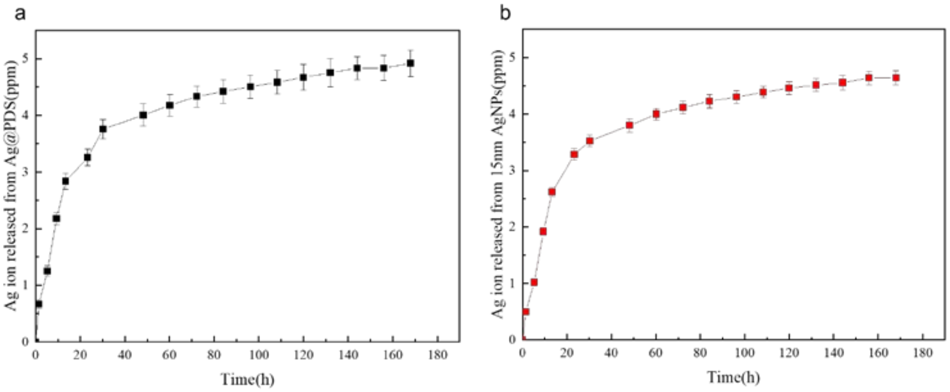

Figure S2. The silver ion released from the Ag@PDS and 15nm AgNPs are merely measurable (< 5ppm) during the observation time with the same Ag concentration.

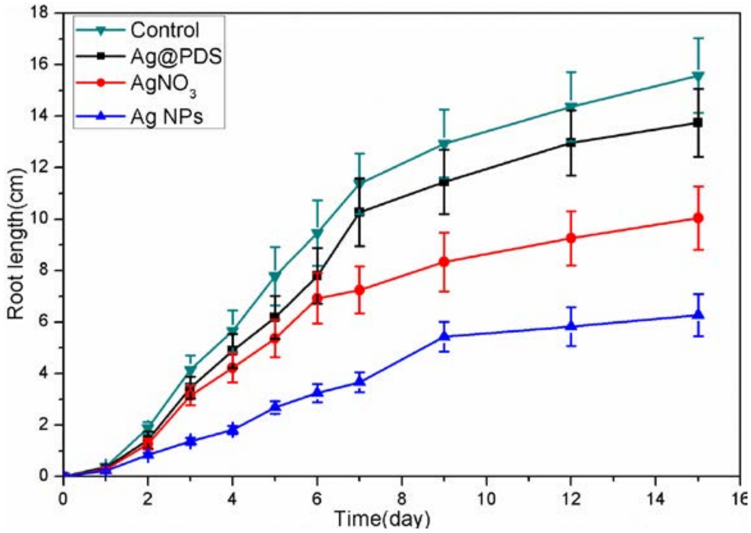

Figure S3. Root length of *Lolium multiflorum* after several days exposure of 40mg/L of Ag@PDS, $AgNO_3$ and Ag NPs.

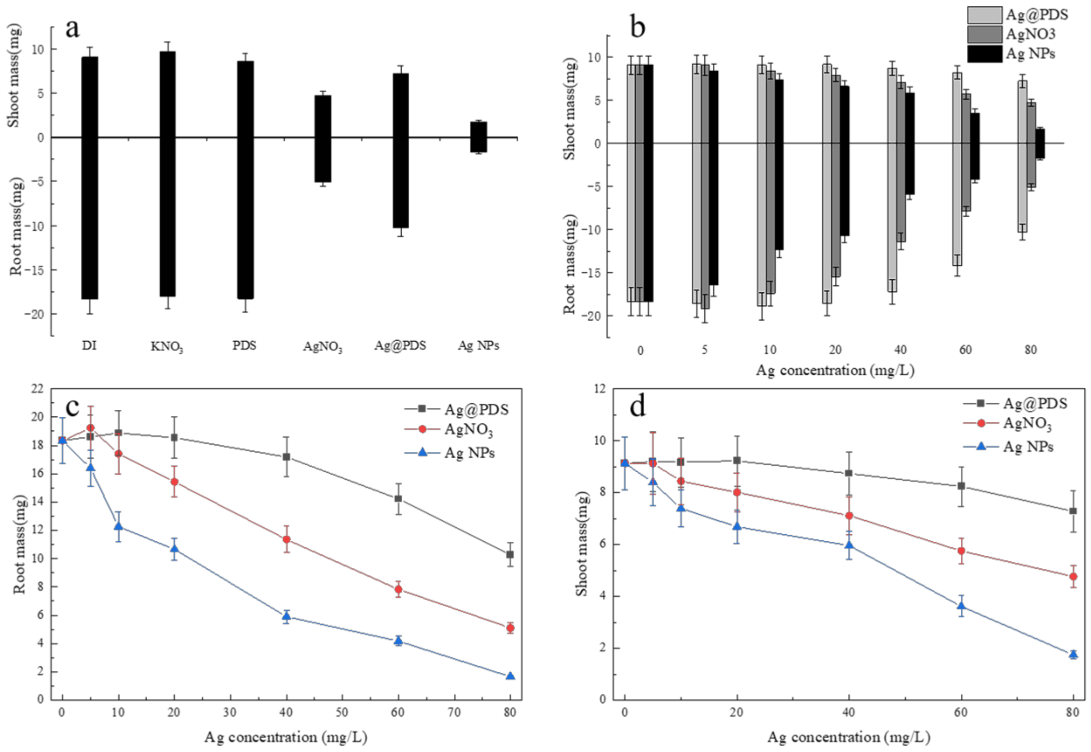

Figure S4. Effect of Ag@PDS, $AgNO_3$ and AgNPs on the *Lolium multiflorum* root (**a, c**) and shoot (**b, d**) mass after 7 days exposure($p < 0.05$).

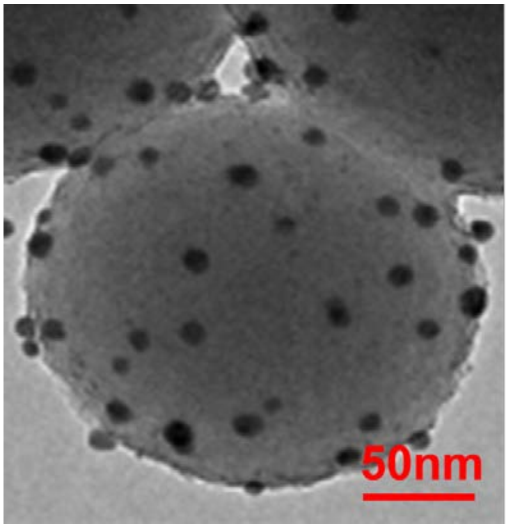

Figure S5. TEM images of poly-dopamine spheres loaded with silver nanoparticles (Ag@PDS) with 50 nm scale bar [1].

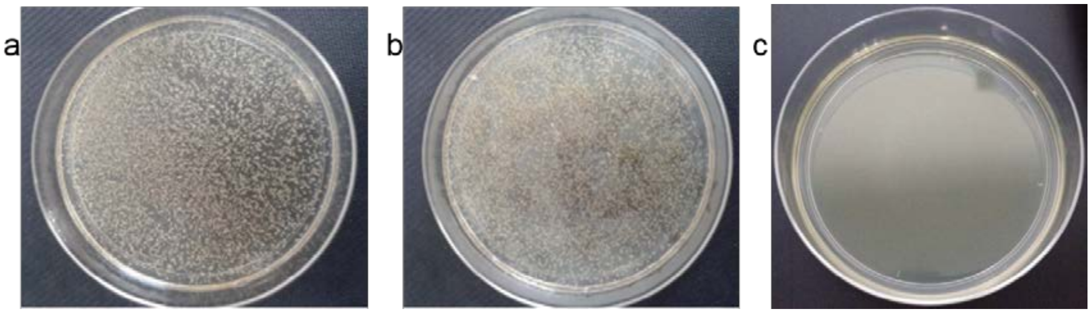

Figure S6. Images of LB-agar plates which is used in the antibacterial activity experiment of samples against E.coli: (**a**)control, (**b**)poly-dopamine(PDS), (**c**) poly-dopamine sphere loaded with silver nanoparticles (Ag@PDS) [1].

References

1. Luo, H. Y., Gu, C. W., Zheng, W. H., Dai, F., Wang, X. L., Zheng, Z., **2015**. Facile synthesis of novel size-controlled antibacterial hybrid sphere with silver nanoparticles loaded to poly-dopamine sphere. RSC Advances, 5: 13470–13477.
